# Metabolomic signatures for the longitudinal reduction of muscle strength over 10 years

**DOI:** 10.1186/s13395-022-00286-9

**Published:** 2022-02-07

**Authors:** Salem Werdyani, Dawn Aitken, Zhiwei Gao, Ming Liu, Edward W. Randell, Proton Rahman, Graeme Jones, Guangju Zhai

**Affiliations:** 1 Division of Biomedical Sciences (Genetics), Faculty of Medicine, Memorial University of Newfoundland, St. John’s, Canada; 2 Menzies Research Institute, University of Tasmania, Hobart, Australia; 3 Division of Community Health and Humanities, Faculty of Medicine, Memorial University of Newfoundland, St. John’s, Canada; 4 Discipline of Laboratory Medicine, Faculty of Medicine, Memorial University of Newfoundland, St. John’s, Canada; 5 Discipline of Medicine, Faculty of Medicine, Memorial University of Newfoundland, St. John’s, Canada

**Keywords:** Muscle strength reduction, Hand grip, Knee extension, Leg muscle strength, Metabolomics, Biomarkers, Asymmetric dimethylarginine, Uric acid

## Abstract

**Background:**

Skeletal muscles are essential components of the neuromuscular skeletal system that have an integral role in the structure and function of the synovial joints which are often affected by osteoarthritis (OA). The aim of this study was to identify the baseline metabolomic signatures for the longitudinal reduction of muscle strength over 10 years in the well-established community-based Tasmanian Older Adult Cohort (TASOAC).

**Methods:**

Study participants were 50–79 year old individuals from the TASOAC. Hand grip, knee extension, and leg strength were measured at baseline, 2.6-, 5-, and 10-year follow-up points. Fasting serum samples were collected at 2.6-year follow-up point, and metabolomic profiling was performed using the TMIC Prime Metabolomics Profiling Assay. Generalized linear mixed effects model was used to identify metabolites that were associated with the reduction in muscle strength over 10 years after controlling for age, sex, and BMI. Significance level was defined at *α*=0.0004 after correction of multiple testing of 129 metabolites with Bonferroni method. Further, a genome-wide association study (GWAS) analysis was performed to explore if genetic factors account for the association between the identified metabolomic markers and the longitudinal reduction of muscle strength over 10 years.

**Results:**

A total of 409 older adults (50% of them females) were included. The mean age was 60.93±6.50 years, and mean BMI was 27.12±4.18 kg/m^2^ at baseline. Muscle strength declined by 0.09 psi, 0.02 kg, and 2.57 kg per year for hand grip, knee extension, and leg strength, respectively. Among the 143 metabolites measured, 129 passed the quality checks and were included in the analysis. We found that the elevated blood level of asymmetric dimethylarginine (ADMA) was associated with the reduction in hand grip (*p*=0.0003) and knee extension strength (*p*=0.008) over 10 years. GWAS analysis found that a SNP rs1125718 adjacent to *WISP1*gene was associated with ADMA levels (*p*=4.39*10^-8^). Further, we found that the increased serum concentration of uric acid was significantly associated with the decline in leg strength over 10 years (*p*=0.0001).

**Conclusion:**

Our results demonstrated that elevated serum ADMA and uric acid at baseline were associated with age-dependent muscle strength reduction. They might be novel targets to prevent muscle strength loss over time.

**Supplementary Information:**

The online version contains supplementary material available at 10.1186/s13395-022-00286-9.

## Background

Skeletal muscles are essential components of the neuromuscular skeletal system [1]. As we get older, loss in skeletal muscle mass and strength increases [2]. Age-related muscle weakness and atrophy, which is called sarcopenia, is one of the earliest signs of aging and is an important geriatric condition [3] that has been associated with several health and socioeconomical consequences [4]. Muscle weakness and wasting leads to a higher risk of falls, fractures [5], loss of function, and disability in older adults [6]. Moreover, loss of muscle mass was found to be associated with an increased loss of medial and lateral tibial cartilage over 2 years [7]. Hip OA patients had lower limb muscle strength and volume deficits [8]. Our recent study on endotypes of OA also showed that muscle weakness indicated by an elevated butyrylcarnitine level might be responsible for a subset of OA patients [9]. Thus, understanding the potential mechanisms of the age-related muscle strength reduction would provide avenue to develop intervention strategies to improve the quality of life in older adults.

Metabolites are the end products of cellular processes that affect or are affected by genetics, lifestyle, and environmental changes. Thereby, their concentrations provide functional information about the physiological state of observed phenotypes [10]. Recent advances in the metabolomic analysis offered new opportunities to measure diverse cell or body fluid metabolites, which have already improved our knowledge about the molecular mechanisms underlying metabolism and the corresponding human traits and diseases [11]. In this study, we investigated the baseline metabolomic signatures in relation to the longitudinal reduction of muscle strength over a 10-year period in a well-established community-based Tasmanian Older Adult Cohort (TASOAC) Study.

## Methods

### Study participants

The study was conducted as part of the TASOAC Study, a prospective, population-based study aimed at identifying the environmental, genetic, and biochemical factors associated with OA [12]. Older adults who were 50–79 years old at recruitment were selected randomly with an equal number of men and women from the roll of electors in southern Tasmania, Australia, and provided a written informed consent [12].

### Demographic information

Demographic, joint symptoms, and daily physical activity information were obtained by a self-administered questionnaire, and anthropometric data including height and weight were measured at clinical interview [12]. Age at baseline was provided and body mass index (BMI) was calculated by dividing weight in kilograms by squared height in meters.

### Muscle strength measurements

Hand grip, knee extension, and leg muscle strength measurements were performed upon baseline, 2.6-, 5-, and 10-year follow-up time points. Hand grip strength was measured using a pneumatic bulb dynamometer (North Coast^TM^ bulb dynamometer; adult 0–30 psi, model no.70154). Participants were seated straight on a chair and held the dynamometer with their elbow at a 90° angle, and their opposite arm resting on their lap [13, 14]. The test was conducted twice for each hand interchangeably, with a 30 seconds rest between trials, and the mean score to the nearest pounds per square inch (psi) was used in the analyses [13].

Knee extension strength of the dominant leg was measured by isometric contraction of knee extensors to the nearest kilogram [14]. Subjects were seated on a custom dynamometer chair having a 100-kg pocket balance connected to the back of the chair with their hips and knees at 90° angle. They were asked to keep their backs straight and grip the chair throughout the test. A strap was placed 10 cm above participants’ lateral malleolus and attached to the dynamometer that recorded maximum contractile force whilst participants attempted to extend their leg. Measurement was taken twice and the average was used in the analysis [14].

Leg muscle strength was measured to the nearest kilogram in both legs simultaneously using a dynamometer (TTM Muscular Metre, Tokyo, Japan) [14]. Study subjects were seated on the back of a dynamometer platform while their knees were flexed by 115° angle, and their backs were rested on a wall [14]. Then, participants were instructed to lift the dynamometer bar to the maximum contractile force, using their legs while their head and neck constant. Measurement was taken twice, then the best reading was used in the analysis [14].

### Metabolic profiling

Blood samples were collected at 2.6-year follow-up point after at least 8 hours fasting, and serum was separated from the blood and stored at −80°C until analysis. We used the samples collected at this time point because the baseline collected samples were depleted. It was the closest time point to the baseline, thus could be considered as baseline surrogate. Targeted metabolic profiling was performed using the TMIC Prime Metabolomics Profiling Assay which quantifies 143 compounds including 40 acylcarnitines, 25 amino acids and derivatives, 23 biogenic amines, one amine oxide, one carboxylic acid, one monosaccharide, 17 organic acids, 34 phospho-and sphingolipids, and one vitamin and cofactor (Supplementary Table 1). The profiling was done at the Metabolomics Innovation Centre (TMIC) using an AB SCIEX QTRAP®4000 mass spectrometer (Sciex Canada, Concord, ON, Canada) equipped with an Agilent 1260 series ultra-high-performance liquid chromatography (UHPLC) system (Agilent Technologies, Palo Alto, CA, USA). The Analyst software 1.6.2 (Concord, ON, Canada) was used to control the entire assay’s workflow, and the metabolite concentrations were reported in μM. The coefficient of variation (CV) for the metabolites ranged between 1.16 and 15.93%.

### Statistical analysis

Metabolites with missing values or with concentration below the limit of detection (LOD) in more than 10% of the samples were removed from the analysis to minimize the false positive results as a standard practice in metabolomics studies [15]. Then, the metabolite concentrations were log transformed and used in the subsequent analysis. The average hand grip strength was calculated from the right- and left-hand grip strength measurements and was used in the analysis. Generalized linear mixed effects model with restricted maximum likelihood (REML) method implemented in R package nlme [16] was used to identify the metabolites that were associated with the longitudinal reduction of hand grip strength, knee extension, and leg muscle strength over 10 years. An interaction term between each metabolite and a follow-up time variable was introduced into the multiple regression models as a predictor for longitudinal changes in the muscle strength, and the beta coefficient for the interaction term was interpreted as the rate of muscle strength change per year over the follow-up time in relation to a given metabolite concentration unit.

Random effects of the y-intercept (sample-ID) and slope (muscle strength reduction over the follow-up time) were utilized to account for the excess variation implicit in the study design between and within study subjects, respectively. The analyses were adjusted for age, sex, and BMI as potential confounders, as shown in the below linear mixed effects model equation of our analyses:

$${\displaystyle \begin{array}{l}\mathrm{library}\left(\mathrm{nlme}\right)\\ {}\mathrm{lme}\left(\mathrm{Muscle}\ \mathrm{Strength}\sim \mathrm{follow}-\mathrm{up}\ \mathrm{time}+\mathrm{Sex}+\mathrm{Age}+\mathrm{BMI}\right.\\ {}+\mathrm{Metabolite}+\left(\mathrm{follow}-\mathrm{up}\ \mathrm{time}\ast \mathrm{Metabolite}\right),\\ {}\mathrm{data}=\mathrm{TASOAC}\_\mathrm{Data},\mathrm{random}=\sim \mathrm{follow}-\mathrm{up}\ \mathrm{time}\mid \mathrm{SampleID},\\ \left.{}\mathrm{control}=\mathrm{list}\left(\mathrm{opt}=``\mathrm{optim}"\right),\mathrm{method}=``\mathrm{REML}",\mathrm{na}.\mathrm{action}=\mathrm{na}.\mathrm{omit}\right)\end{array}}$$

Significance level was defined at *α*=0.0004 after correcting for multiple testing of 129 metabolites with Bonferroni method.

Since the metabolomics profiling was conducted at the 2.6-year follow-up point only, and the age range of the study participant spanned for ~30 years, a multiple linear regression was performed to investigate the cross-sectional association of the identified metabolites with the muscle strength measurements at the 2.6-year follow-up phase.

Further, a genome-wide association analysis (GWAS) was performed on 77 individuals from the Newfoundland Osteoarthritis Study (NFOAS) whose metabolomic and GWAS data were available from previous studies [9, 17]. The GWAS analysis was conducted to explore the potential mechanisms of the association between the metabolomic markers and the longitudinal reduction of muscle strength over 10 years using the commonly accepted GWAS significance threshold *p* < 5*10^−8^.

## Results

**Fig. 1:**
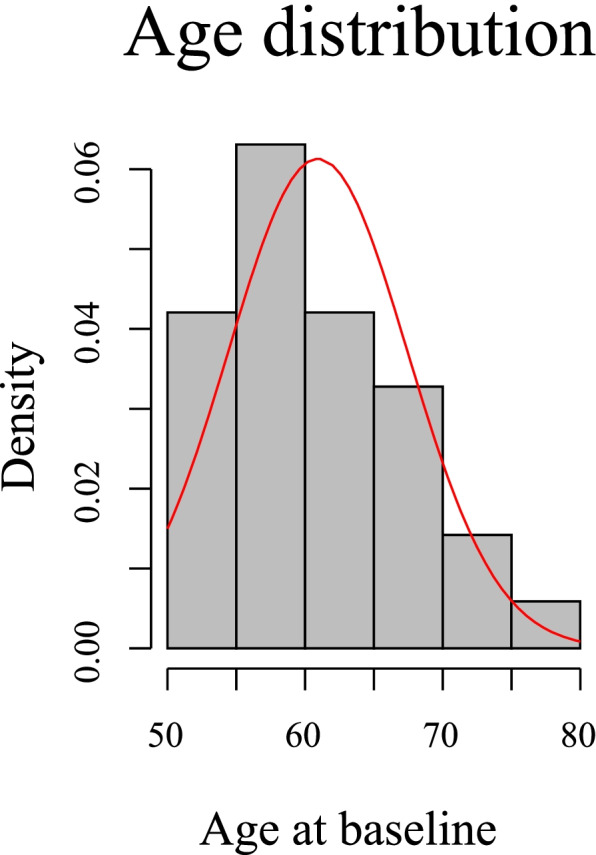
A histogram showing the 409 study participants age distribution at baseline

A total of 409 subjects (50% of them females) were included in this study. The subjects were followed up for three time phases with the mean follow-up time of 2.60±0.40, 5.06±0.48, and 10.73±0.67 years, respectively. The mean age was 60.93±6.50 years (Fig. 1), and mean BMI was 27.12±4.18 kg/m^2^ at baseline. Males were older than females (*p*=0.02), but there was no significant differences in BMI between males and females at baseline, as well as at each of the follow-up phases (Table 1). Further, BMI did not change significantly over 10 years for both sexes (*p*=0.06).

**Table 1:** The characteristics of the study participants (*n*=409)

	Male	Female	***P*** value
**Sex (number (%))**	205 (50.12)	204 (49.88)	1
**Age (years)**	64.27±6.91	62.79±5.94	0.02
**Baseline BMI (kg/m**^**2**^**)**	27.12±3.54	27.12±4.74	0.99
**2.6-year BMI (kg/m**^**2**^**)**	27.18±3.64	28.59±16.99	0.25
**5-year BMI (kg/m**^**2**^**)**	27.44±3.70	28.96±17.29	0.22
**10-year BMI (kg/m**^**2**^**)**	27.57±4.10	27.64±5.44	0.87

Values are mean ± SD for continuous variables and percentage for sex. *P* values were obtained from chi-squared test for sex distribution and Student’s *t* test for continuous variables. *BMI* body mass index

Similar to other longitudinal analysis, our study had few individuals that did not complete all four follow-up muscle strength measurements. Table 2 lists the numbers and percentages of the study participants that completed the hand grip, knee extension, and leg muscle strength measurements at the baseline and each of the follow-up time points.

**Table 2:** The number and percentage of the study participants that completed the muscle strength measurements at each of the follow-up time points

	Baseline number (%)	2.6-year-point number (%)	5-year-point number (%)	10-year-point number (%)
**Hand grip**	409 (100)	408 (99.76)	408 (99.76)	407 (99.51)
**Knee extension**	408 (99.76)	407 (99.51)	407 (99.51)	403 (98.53)
**Leg strength**	399 (97.56)	396 (96.82)	386 (94.38)	377 (92.18)

**Fig. 2:**
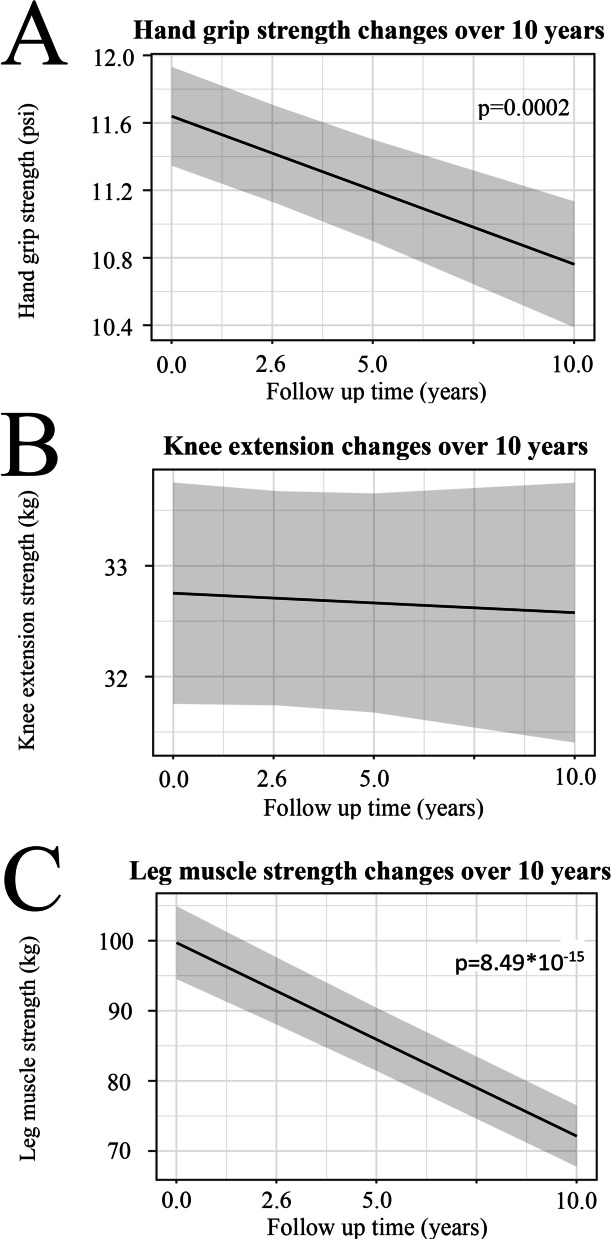
Plots of the fitted lines for changes over 10-year follow-up time for **A** hand grip strength, **B** knee extension, and **C** leg muscle strength estimated by linear mixed regression model done with nlme R package with the function lme(Muscle Strength ~ follow-up time, data=TASOAC_Data, random=~ follow-up time|SampleID, control=list(opt = “optim”), method= “REML”, na.action=na.omit)**.** The gray shaded areas are 95% CI

Our data showed that hand grip declined by 0.09 psi per year (*p*=0.0002), and leg muscle strength decreased by 2.57 kg per year (*p*=8.49*10^-15^), while the decrease in knee extension of 0.02 kg per year was not statistically significant (*p*=0.58), estimated by the generalized linear mixed affects model (Fig. 2, Table 3).

**Table 3:** Muscle strength differences in the whole cohort (*n*=409), and between males and females over 10-year follow-up period

		Whole dataset	Male	Female	Male VS female ***P*** value
**Hand grip** (psi)	**Baseline**	12.3±2.99	14.4±2.53	10.3±1.77	2.76*10^-14^
	**2.6-years**	10.1±2.93	11.9±2.59	8.43±2.15	1.03*10^-4^
	**5-years**	11.7±4.35	13.9±4.36	9.37±2.92	2.13*10^-5^
	**10-years**	10.8±3.64	12.8±3.62	8.91±2.45	3.12*10^-4^
**Decline/year** (psi/year)		0.09	0.09	0.09	
***P*** **value**		0.0002	1.2*10^-14^	1.46*10^-14^	
**Knee extension** (kg)	**Baseline**	31.55±11.3	38.2±9.19	24.8±9.12	2.74*10^-18^
	**2.6-years**	33.2±11.0	38.8±9.17	27.6±9.71	2.36*10^-12^
	**5-years**	34.3±10.3	39.5±8.69	29.2±9.2	1.52*10^-14^
	**10-years**	31.54±11.3	38.5±12.6	24.7±9.32	8.28*10^-7^
**Decline/year** (kg/year)		0.02	0.002	0.067	
***P*** **value**		0.58	0.50	0.20	
**Leg strength** (kg)	**Baseline**	96.0±52.6	132±46.3	59.6±27.7	1.82*10^-4^
	**2.6-years**	96.7±53.4	133±46.5	60.1±29.5	5.36*10^-4^
	**5-years**	87.3±54.4	124±47.6	50.1±30.1	3.48*10^-5^
	**10-years**	70.4±43.9	95.3±43.6	45.4±26.8	3.71*10^-4^
**Decline/year (**Kg/year)		2.57	3.68	1.47	
***P*** **value**		8.49*10^-15^	2.0*10^-16^	2.09*10^-8^	

Values are mean ± SD for muscle strength measurement at baseline, 2.6-, 5-, and 10-year follow-up phases. *P* values for muscle strength differences between males and females at each follow-up points were obtained by using the linear regression (lm) method in *R*; *psi* pounds per square inch, *kg* kilogram

**Fig. 3:**
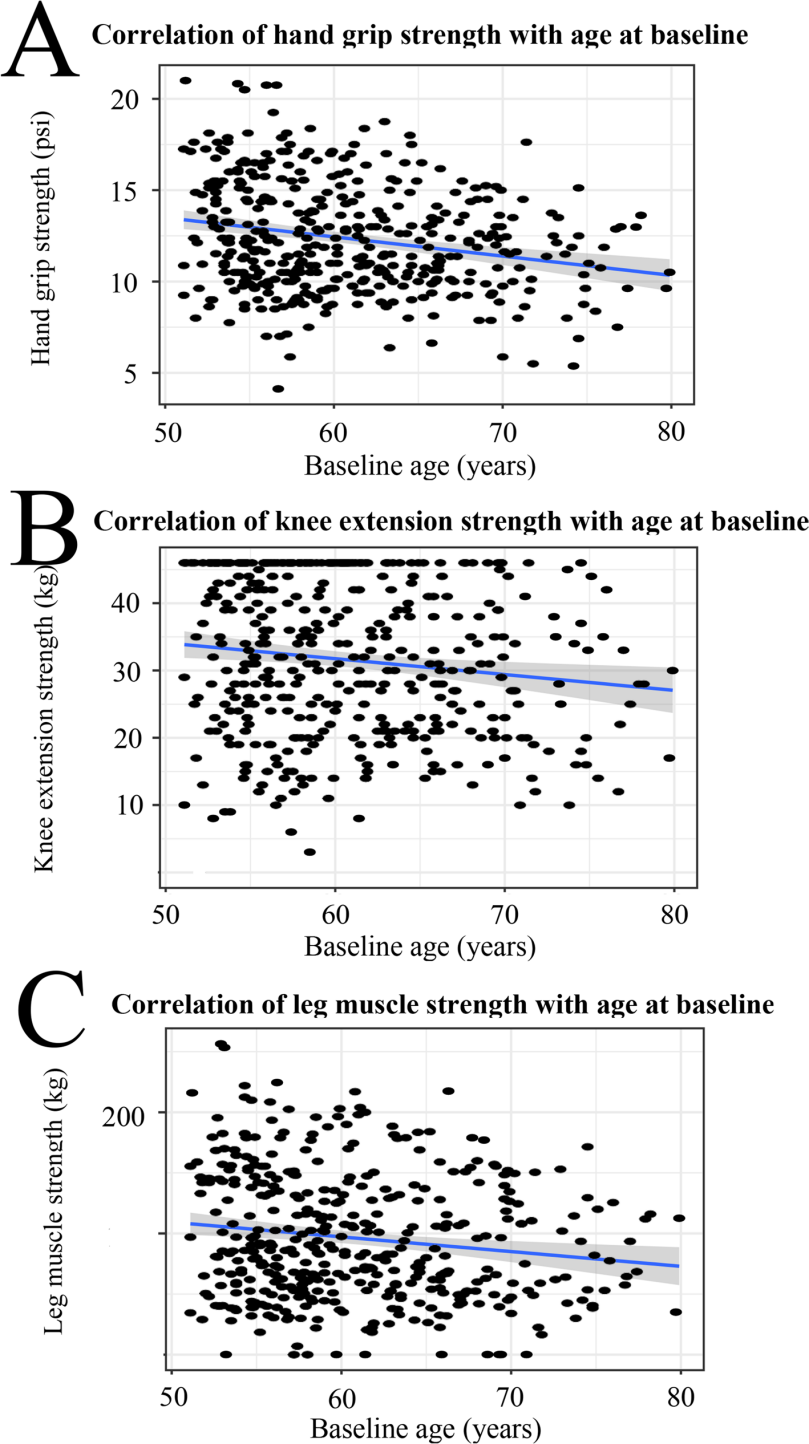
The relationship between muscle strength and the study participants’ age at the baseline, **A** hand grip strength, **B** knee extension strength, and **C** leg muscle strength changes

Furthermore, our findings showed a significant association between higher age and lower hand grip (*p*=1*10^-15^), knee extension (*p*=4.5*10^-15^), and leg muscle strength (*p*=3.4*10^-12^) at the baseline (Fig. 3).

**Fig. 4:**
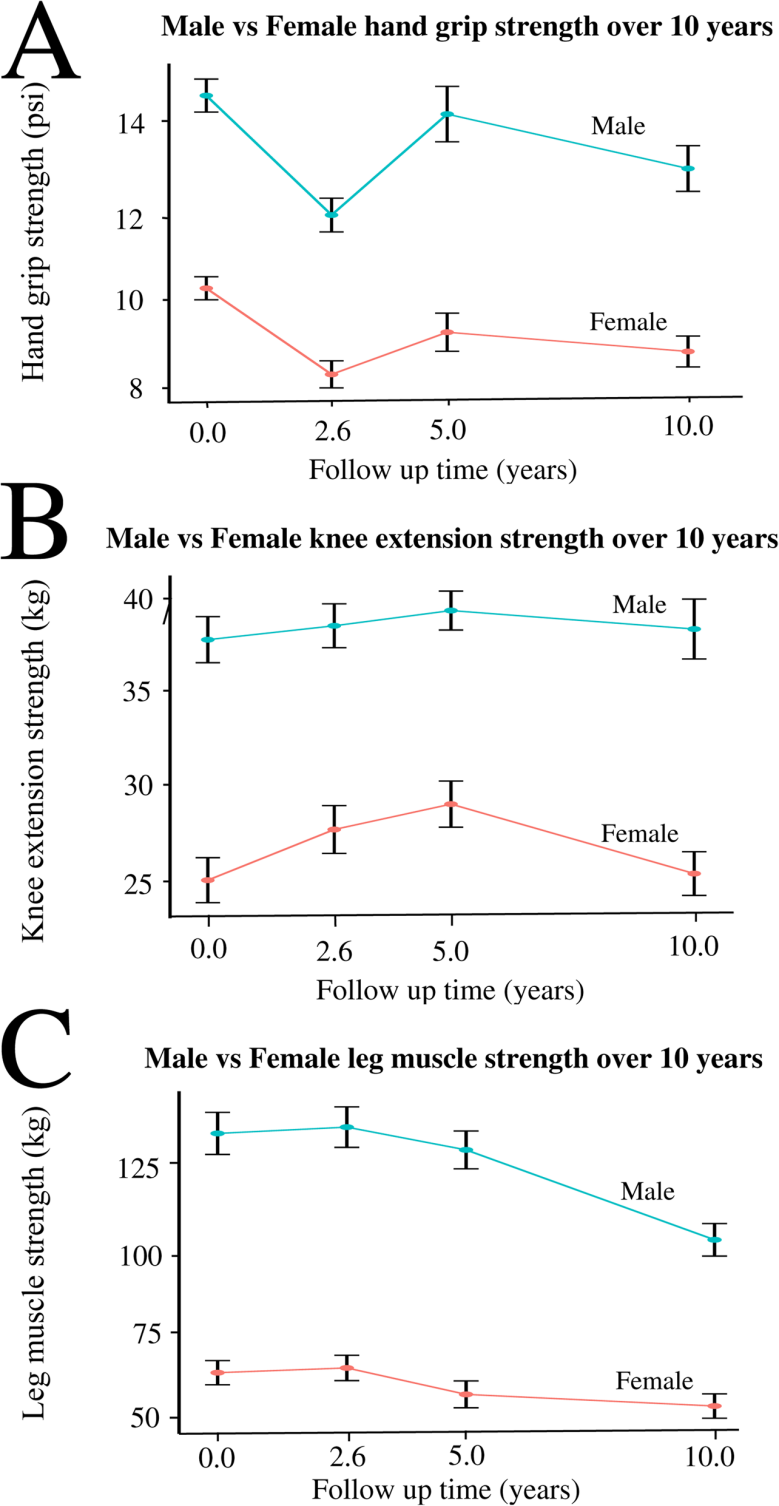
The differences between males and females in muscle strength changes over 10-year follow-up time **A** hand grip strength, **B** knee extension strength, and **C** leg muscle strength. *Bars represent 95% CI

Although all muscle strength measurements in males were significantly higher than that in females over the 10-year follow-up time (*p*=2*10^-15^), at the baseline, and at each of the follow-up phase (all *p*=5.36*10^-4^, Table 3), there was no significant difference (*p*=0.24) in the muscle strength change rate between males and females over the 10-year follow-up period (Fig. 4). Interestingly, higher BMI in the 409 study participants was associated with higher knee extension strength (*p*=0.036).

**Fig. 5:**
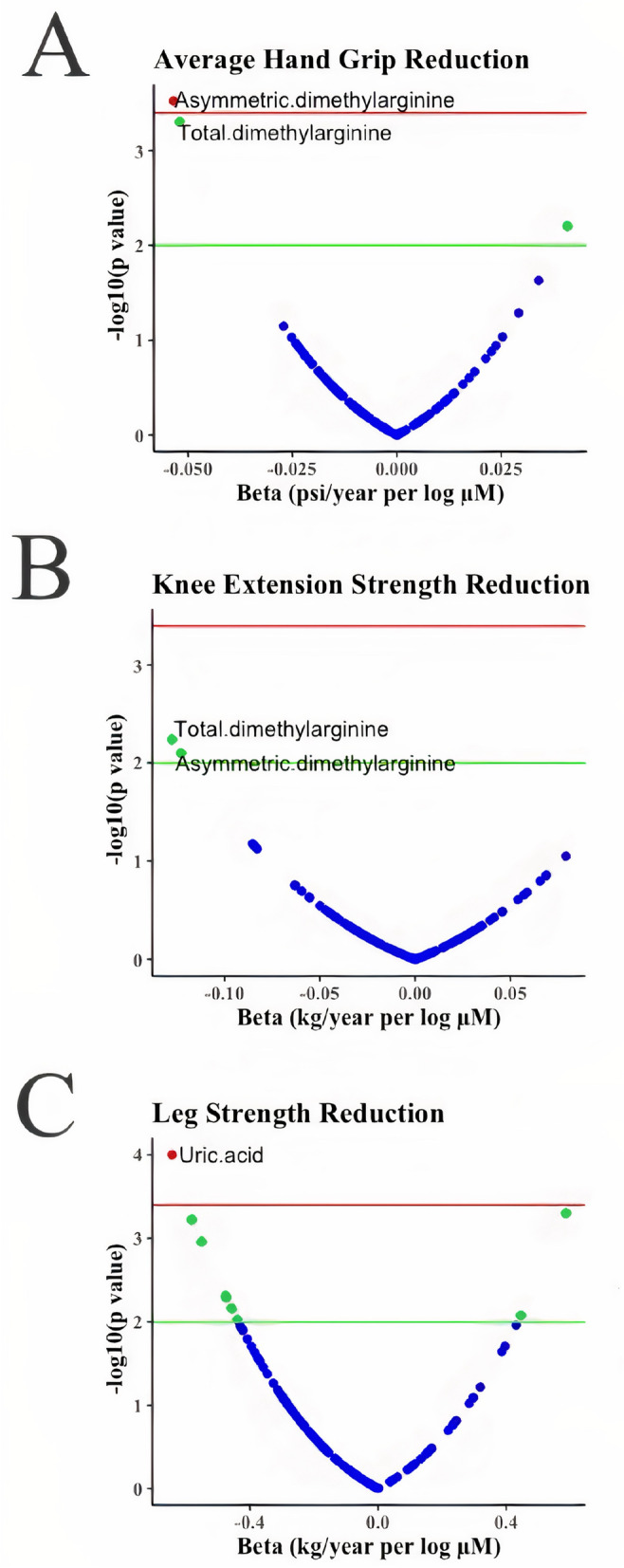
Volcano plots of the association results between metabolites and the changes of muscle strength: **A** hand grip strength change, **B** knee extension strength change, and **C** leg muscle strength change over 10-year follow-up time. *X*-axis is the effect size in betas obtained from the generalized linear mixed effects model, and *y*-axis is minus log transformed *p* values. The green line indicates *p* value = 0.01, and the red line shows the pre-defined significance level at *α*=0.0004 after correction of multiple testing of 129 metabolites with Bonferroni method. A negative value of beta in the *X*-axis refers to a reduction in muscle strength per unit increase for a given metabolite, while a positive value of beta in the *X*-axes refers to an increase in muscle strength per unit increase for a given metabolite over the 10-year follow-up time

Among the 143 metabolites measured, 129 passed the quality checks and were included in the subsequent analysis (Supplementary Table 2). The volcano plot in Fig. 5A shows the results of the association between the reduction of the hand grip strength and each of the 129 metabolites. While four metabolites had *p*<0.05, only one metabolite—asymmetric dimethylarginine (ADMA) was associated with the reduction in the hand grip strength at the pre-defined significance level (*p*=0.0003). Per log μM increase in ADMA was associated with 0.05±0.02 psi/year reduction rate in the hand grip strength (Table 4). The total dimethylarginine was the second top metabolite associated with the hand grip strength reduction, but the *p* value (*p*=0.0006) did not reach the pre-defined significance (Fig. 5A). Per log μM increase of this metabolite was associated with 0.05±0.01 psi/year reduction rate in the hand grip strength (Table 4). Taurine (beta=0.03±0.01 psi/year per log μM; *p*=0.015) and lactic acid (beta=0.03±0.01 psi/year per log μM; *p*=0.04) were also potentially associated with the reduction in hand grip strength over 10 years (Table 4).

**Table 4:** Top metabolites associated with the muscle strength change measures in the study participants (*n*=409) over 10-year follow-up time

Muscle strength change	(Metabolite*follow-up time)^a^	Beta	SE	***P*** value
A) **Hand grip**	Asymmetric dimethylarginine	−0.053	0.015	0.0003
	Total dimethylarginine	−0.049	0.014	0.0006
	Taurine	0.033	0.014	0.015
	Lactic acid	0.029	0.014	0.04
B) **Knee extension**	Total dimethylarginine	−0.119	0.044	0.007
	Asymmetric dimethylarginine	−0.119	0.045	0.008
	Methylmalonic acid	−0.085	0.046	0.07
	C3:1	−0.084	0.046	0.07
C) **Leg strength**	Uric acid	−0.633	0.157	0.0001
	PC aa 32:2	0.586	0.168	0.0005
	Creatinine	−0.581	0.168	0.0006
	Methionine	−0.551	0.168	0.001

^a^An interaction term between metabolite values and follow-up time used as predictors for longitudinal changes in muscle strength. *C3:1* acrylylcarnitine, *PC aa 32:2* phosphatidylcholine acyl-acyl with 32 carbons and 2 double bonds

The volcano plot in Fig. 5B presents the results of the association between the change of knee extension strength over time and each of the 129 metabolites. While total dimethylarginine and ADMA were the top associated metabolites with the reduction of knee extension with *p*<0.05 level, both of them did not reach the pre-defined significance level (Table 4).

The volcano plot in Fi. 5C presents the results of the association between the reduction in leg muscle strength and each of the 129 metabolites. A total of 24 metabolites were significantly associated with the reduction in leg muscle strength at *p*<0.05 level, but uric acid was the only metabolite that reached the pre-defined significance (*p*=0.0001). Per log μM increase in uric acid was associated with 0.63±0.16 kg/year reduction in leg muscle strength (Table 4). We also tested the interaction between sex and uric acid which was not statistically significant (*p*=0.83). In addition, diacyl-phosphatidylcholines with 32 carbons and two double bonds (PC aa C32:2; beta=0.59±0.17 kg/year per log μM; *p*=0.0005), creatinine (beta=−0.58±0.17 kg/year per log μM; *p*=0.0006), and methionine (beta=−0.55±0.17 kg/year per log μM; *p*=0.001) were potentially associated with the reduction rate of leg muscle strength over 10-year follow-up period (Table 4).

Further, the increased serum concentration of ADMA and total dimethylarginine were associated with difficulty in run errands and shopping (*p*≤0.008), vacuuming (*p*≤0.001), and bathing (*p*=0.0003). The elevated uric acid level was associated with hardship of putting on socks (*p*=0.04), and climbing up five steps (*p*=0.046) over 10-year follow-up period.

Since the metabolomic profiling was performed on the serum samples collected at 2.6-year follow-up point, we performed a cross-sectional association test for the visit at 2.6 years and found that the higher levels of ADMA (*p*=0.027) and total dimethylarginine (*p*=0.01) were associated with a lower knee extension, but not with hand grip strength (all *p*=0.42). There was no cross-sectional association between uric acid and leg muscle strength (*p*=0.56) at the 2.6-year follow-up point.

Further, we dividied the cohort into three different age groups based on the sample size distribution: younger (*n*=140), middle age (*n*=146), and older (*n*=123) groups and examined whether the identified metabolite associations were stronger in the older group. The results showed that the effect size of ADMA and total dimethylarginine on hand grip and knee extension in the older age group was greater than that in the younger groups. The effect size of the uric acid on leg strength was larger in the middle age group than the younger and the older groups (Supplementary Table 3).

**Fig. 6:**
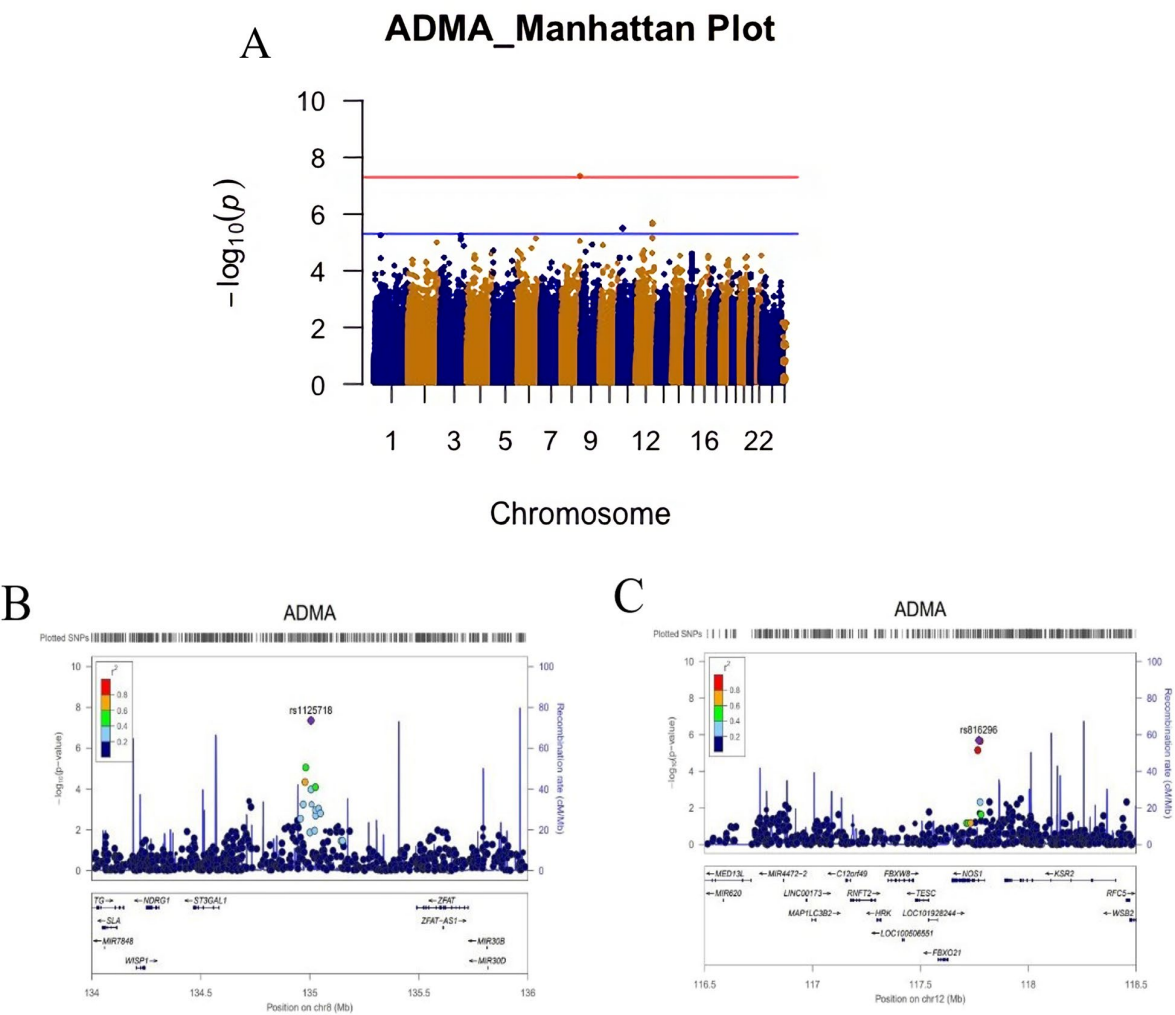
Genome wide association study (GWAS) for the asymmetric dimethylarginine (ADMA) in the 77 OA patients from the NFOAS: **A** a Manhattan plot of the GWAS results. The red line indicates the GWAS significance at *α* = 5 × 10^−8^, **B** the regional association plot for the chromosomal region around the most significant SNP rs1125718 on chromosome 8 (*p*=4.394*10^-8^) showing adjacent *NDRG1*, *WISP1*, *ST3GAL1*, and *ZFAT* genes, and **C** the regional association plot for the chromosomal region around the second most significant SNP rs816296 on chromosome 12 (*p*=2.03*10^-6^). This SNP is an intronic variant located in intron 1 of *NOS1* gene

The GWAS analysis was performed on the metabolites that reached the pre-defined significance level. Figure 6A shows the Manhattan plot of the GWAS results for ADMA. We found that a single nucleotide polymorphism (SNP) rs1125718 (G>A, with minor allele frequency (MAF=0.29)) on chromosome 8 was associated with elevated ADMA concentrations at GWAS significance level (*p*=4.39*10^-8^). This SNP is located in the gene desert on chromosome 8, but adjacent to several genes including N-Myc Downstream Regulated 1 (*NDRG1*), WNT1 Inducible Signaling Pathway Protein 1(*WISP*1), ST3 Beta-Galactoside Alpha-2,3-Sialyltransferase 1 (*ST3GAL1*), and Zinc Finger And AT-Hook Domain Containing (*ZFAT*) (Fig. 6B). Although it did not reach the GWAS significance, the second most associated SNP rs816296 (C>A, MAF=0.17, *p*=2.03*10^-6^) on chromosome 12 is located in intron 1 of the Nitric Oxide Synthase 1 (*NOS1*) gene (Fig. 6C). Data on uric acid was not available in the NFOAS; thus, no GWAS analysis was performed for uric acid.

## Discussion

To the best of our knowledge, this was the first population-based study that investigated the relationship between the serum metabolome and the longitudinal reduction rates in hand grip, knee extension, and leg muscle strength over 10-year follow-up period in a large sample size of older adults that were randomly selected from a general population. The reduction rate of the hand grip and leg muscle strength in the current study were comparable with previous studies [18–20], but the reduction rate of the knee extension was lower in the current study than the previous report [21], which might be due to the difference in study populations, follow-up times, study designs, and measurement methods.

We reported that elevated serum concentrations of dimethylarginines, especially ADMA, were significantly associated with the longitudinal reduction rate in the hand grip and knee extension strength. Interestingly, the elevated ADMA and total dimethylarginine blood levels were also associated with functional impairments including run errands and shopping, vacuuming, and bathing over the 10-year follow-up period. We also found that elevated uric acid concentration was significantly associated with the decline rate in leg muscle strength over a 10-year follow-up period. The increased uric acid level was also associated with the longitudinal complication of putting on socks, and climbing up five steps over 10-year follow-up period.

However, there is still much more in common between a leg muscle strength and a knee extension, than between hand grip and knee extension, it is interesting that both hand grip and knee extension had negative correlation with ADMA and total dimethylarginine blood levels, while leg muscle strength decline had a significant association with the elevated blood level of uric acid. This might be due to the hand grip and knee extension common functionalities and mechanisms. Wrist and finger flexion are mostly initiated by the muscles in the anterior and posterior compartments of the forearm (extrinsic muscles), and only the thin tendons of these muscles are found directly in the hand. The flexor tendons of the forearm anterior compartments run in the anterior of the hand through the palms to the tips of the fingers to facilitate flexing of the wrist and fingers leading to wrist flexion and hand grip force production [22]. Moreover, the extensor tendons of the forearm posterior compartments used for wrist extension and hand grip relaxation run through the back of the hand to the figures [23]. While the extrinsic muscles of the hand are responsible for stronger movements of the wrist and hand, the intrinsic muscles of the hand have no direct effect on wrist action but can contribute to grip force via the extensor mechanism [24]. The intrinsic muscles produce finer, more controlled movements and play important roles in rotating fingers toward the palm to maintain and improve hand grip [25]. Thus, hand grip strength is relatively specific for the muscles in the anterior compartment of the forearm that are involved in finger/wrist flexion. Similarly, while the quadriceps femoris in the anterior compartment of the thigh are activated to extend the knee in the knee extension strength test, the hamstrings in the posterior compartment of the thigh are predominantly involved to flex the knee [26]. Also, while the hand grip and knee extension tests are mostly used to assess the upper and lower body’s muscle strength and power, leg muscle strength test is implemented to evaluate the body balance and risk of fall in older adults, because balance consists of multiple body systems including the ability to align different body segments and to generate multi-joint movements to effectively control body position and movement [27]. Morover, As neural decrements present earlier than loss of strength, this would likely affect more complex movements more drastically than measures of specific, relatively isolated muscle groups performed in a stable setting. Indeed, the change in leg strength was more pronounced than grip strength.

Data on ADMA and muscle strength are sparse in the literature. In the cross-sectional study of 550 individuals [28], high serum level of ADMA was associated with lower hand grip, quadriceps strengths, and slower gait speed [28]. Cancer patients [29] were found to have higher levels of ADMA in the skeletal muscle compared with healthy controls, suggesting that increased levels of ADMA may contribute to impaired muscle protein synthesis in cancer cachexia. In the longitudinal setting, our data documented that the elevated ADMA level was associated with the reduction of muscle strength over time, especially hand grip strength and knee extension. Further studies to investigate the causal relationship between ADMA and muscle strength reduction is warranted. The increased blood concentration of the total dimethylarginine was also associated with the strength reduction in the hand grip and knee extension over the follow-up period. However, the effect size was similar to that of ADMA, suggesting that the association was most likely driven by ADMA rather than symmetric dimethylarginine (SDMA).

Dimethylarginines are products of degraded methylated proteins. Two enzymes—protein arginine methyltransferase type I and II (*PRMT-I, PRMT-II*), are involved in the methylation of arginine residues within proteins or polypeptides with the methyl groups derived from *S*-adenosylmethionine [30]. PRMT-I catalyzes the formation of NG-monomethyl-l-arginine (LNMMA) and NG,NG-dimethyl-l-arginine (ADMA) while PRMT-II methylate proteins to release NG,N’G-dimethyl-l-arginine (SDMA) and LNMMA. Free dimethylarginines are released into the cytoplasm during proteolytic breakdown of proteins and can be detected in blood, and eliminated from the body by renal excretion [31]. ADMA, but not SDMA, is metabolized via hydrolytic degradation to citrulline and dimethylamine by the dimethylarginine dimethylaminohydrolase-1 (DDAH-1) and -2 (DDAH-2) enzymes. Thus, the increased ADMA levels could be due to increased PRMT-I activity, reduced elimination by the kidney, decreased DDAH-1 and 2 enzymtic activities, or a combination. However, our GWAS analysis did not find any association between ADMA and these genes including PRMT-I and DDAH-1 and 2, suggesting that the increased ADMA level may not be genetic. Instead, we found that SNP rs1125718 on chromosome 8 was associated with ADMA concentration at GWAS significance level. This SNP is located in a gene desert and has not been reported to be associated with any disease or traits yet. However, several genes are located in the nearby region including *NDRG1*, *WISP1*, *ST3GAL1*, and *ZFAT*. Among them, *WISP1* gene is interesting because a study showed that *WISP1* as fibro-adipogenic progenitor (FAP)-derived matricellular signal is lost during aging. WISP1 is required for efficient muscle regeneration, and it controls the expansion and asymmetric commitment of tissue-resident muscle stem cells (MuSCs) through Akt signaling [32]. Also, previous studies showed that nitric oxide (NO) level positively correlated with *WISP1* gene expression, and elevated levels of NO increased the *WISP1* mRNA and protein expression levels through a beta-catenin signaling [33]. Interestingly, ADMA is known as an endogenous competitive inhibitor of NO synthase [34]. Our GWAS analysis showed that the second most associated SNP with ADMA was rs816296 which is located in the intron 1 of the *NOS1* gene. Thus, we hypothesize that possible age-related muscle protein breakdown may lead to an increased release of ADMA which in turn inhibits NO production. The reduced NO synthesis may result in lower expression of WISP1 which leads to the matricellular signals in the skeletal muscle stem cell niche being disturbed [32, 35]. Hence, the MuSC number, activity, adhesion, migration, proliferation, self-renewal, and differentiation in skeletal muscle regeneration could be considerably deteriorated leading to the reduction of muscle strength [36–38].

Uric acid is an enzymatic waste endproduct from the degradation of purine nucleosides that is renally excreted. Uric acid plays both protective and damaging roles in the skeletal muscles [39], most likely due to its strong antioxidant properties at low levels and pro-inflammatory effect at high levels [35]. It has been proposed that oxidative stress might contribute to muscle weakness and wasting. Uric acid at a low level might stabilize the excessive production of free radicals that causes muscle protein damage leading to muscle strength reduction [40]. However, at high levels, uric acid stimulates the pro-inflammatory pathway and elevates the production of pro-inflammatory cytokines including interleukin-1 (IL-1), IL-6, and the tumor necrosis factor (TNF), which have an impact on muscle mass and function in aged muscles [41, 42]. While we did not find a significant cross-sectional association between uric acid and leg muscle strength (*p*=0.56) at the 2.6-year follow-up phase, we found that there was a positive association between uric acid concentrations and leg muscle strength at the baseline time point. This is consistent with previous studies [39, 40]. We also documented a negative association between uric acid levels and longitudinal leg muscle strength, consistent with previous studies [41, 42]. Thus, our findings suggested the importance of maintaining optimal levels of uric acid in the blood for muscle strength [40].

The strength of the current study was its longitudinal nature which allowed us to detect significant metabolite associations for muscle strength changes overtime within an individual. This can not be achieved in a cross-sectional analysis. Indeed, when we analyzed the data cross-setionally for the 2.6-year follow-up point, the significance for the identified metabolites became weaker or even non-significant. The current study also underscored the importance of the longer follow-up time with multiple time point measurements as it could minimize the effect of fluctuating variability on the measurements and provide more accurate estimate of changes over time. However, there are a number of limitations in this study. The present study used a commercially available metabolomics assay kit that offers limited coverage of metabolome. Thus, we might miss some metabolites that may contribute to the longitudinal reduction of muscle strength. Since metabolomics profiling was performed at only 2.6-year follow-up point, we cannot make any inference regarding the association between the changes in metabolite profiles over time and the muscle strength decline over time. Further studies with multiple time point metabolomic profilings are needed. Loss to follow-up might have influenced our results, especially for leg muscle strength as we had 6–8% of missing values at phase 3 and phase 4 follow-up points. Indeed, those lost to follow-up had a lower leg muscle strength measurement at baseline than those included in the analysis (data not shown). However, there was no difference in uric acid levels between those included and excluded in the final analysis, suggesting that loss to follow-up was unlikely to bias the observed association. We cannot rule out the potential confounding effect of gout on the association between uric acid and leg muscle strength as we did not have data on gout in our cohort. However, gout mostly affects big toes and associated with reduced muscle strength of the ankle and foot, not leg muscle strength, suggesting the observed association was less likely to be biased. Finally, our results may not be generalized to populations that have different area-specific socioeconomic indexes and health provisions than that in Tasmania, Australia.

In conclusion, our data demonstrates that baseline elevated serum concentrations of ADMA and uric acid were associated with age-dependent muscle strength reduction. Confirmation of these findings would establish new insights into the pathogenesis of age-related muscle strength decline and uncover novel targets for developing strategies to prevent muscle strength loss over time.

## Supplementary Information

**Additional file 1: Supplementary table 1:** TMIC Prime Metabolomics Profiling Assay list of 143 metabolite concentrations.

**Additional file 2: Supplementary table 2:** Summary statistics for the 129 metabolites that passed the quality checks and were included in the analysis.

**Additional file 3: Supplementary table 3:** Top metabolites associated with the muscle strength change measures in three different age groups over 10-years follow up time.

## Data Availability

The datasets generated and/or analyzed during the current study are not publicly available but are available from the corresponding author on reasonable request.

## Abbreviations

ADMA: Asymmetric dimethylarginine NG NG-dimethyl-l-arginine
BMI: Body mass index
CV: The coefficient of variation
DDAH-1: Dimethylarginine dimethylaminohydrolase-1
DDAH-2: Dimethylarginine dimethylaminohydrolase-2
FAPs: Fibro-adipogenic progenitors
GWAS: Genome-wide association study
IL-1: Interleukin-1
IL-6: Interleukin-6
kg: Kilogram
LNMMA: NG-monomethyl-l-arginine
LOD: Limit of detection
MuSCs: Muscle stem cells
NDRG1: N-Myc Downstream Regulated 1
NFOAS: Newfoundland Osteoarthritis Study
NO: Nitric oxide
NOS1: Nitric oxide synthase 1
OA: Osteoarthritis
PRMT-I: Protein arginine methyltransferase type I
PRMT-II: Protein arginine methyltransferase type II
PSI: Pounds per square inch
REML: Restricted maximum likelihood
SDMA: Symmetric dimethylarginine
NG: N’G-dimethyl-l-arginine
SNP: Single-nucleotide polymorphism
ST3GAL1: ST3 Beta-Galactoside Alpha-2,3-Sialyltransferase 1
TASOAC: Tasmanian Older Adult Cohort
TMIC: The Matabolomic Innovation Centre
TNF: Tumor necrosis factor
UHPLC: Ultra-high-performance liquid chromatography system
WISP1: WNT1 Inducible Signaling Pathway Protein 1
ZFAT: Zinc Finger And AT-Hook Domain Containing
