# Supplementary material for: Metabolomic signatures for the longitudinal reduction of muscle strength over 10 years
Source: Skelet Muscle. 2022 Feb 7;12:4. doi: 10.1186/s13395-022-00286-9 (PMC8819943; doi:10.1186/s13395-022-00286-9)
Supplement: Supplementary file 1 — Additional file 1: Supplementary table 1: TMIC Prime Metabolomics Profiling Assay list of 143 metabolite concentrations. [file 13395_2022_286_MOESM1_ESM.docx]

**Supplementary table 1:** TMIC Prime Metabolomics Profiling Assay list of 143 metabolite concentrations.

| **Metabolite class** | **Number** | **Metabolite name or abbreviation** |
| --- | --- | --- |
| Carnitine | 1 | C0 |
| Acylcarnitine | 25 | C2, C3, C3:1, C4, C4:1, C5, C5:1, C6(or C4:1-DC), C6:1, C8, C9, C10, C10:1, C10:2, C12, C12:1, C14, C14:1, C14:2, C16, C16:1, C16:2, C18, C18:1, C18:2 |
| Hydroxy- and dicarboxyacylcarnitines | 14 | C3-OH, C4-OH(or C3-DC), C5:1-DC, C5-DC(or C6-OH), C5-M-DC, C5-OH(or C3-DC-M), C7-DC, C12-DC, C14:1-OH, C14:2-OH, C16:1-OH, C16:2-OH, C16-OH, C18:1-OH |
| Amino Acids | 22 | Alanine, Arginine, Asparagine, Aspartate, Citrulline, Glutamine, Glutamate, Glycine, Histidine, Isoleucine, Leucine, Lysine, Methionine, Ornithine, Phenylalanine, Proline, Serine, Threonine, Tryptophan,Tyrosine, Valine, Betaine |
| Amino Acid Derivatives | 3 | Creatine, Phosphocreatine, Methylhistidine |
| Biogenic Amines | 23 | Acetylornithine, Asymmetric dimethylarginine, Total dimethylarginine, Alpha-Aminoadipic acid, Carnosine, Creatinine, Dihydroxyphenylalanine (DOPA), Dopamine, Histamine, Kynurenine, Methioninesulfoxide, Hydroxyproline (c4-OH-Pro), Hydroxyproline (t4-OH-Pro), Nitrotyrosine, Phenylethylamine, Putrescine, Sarcosine, Serotonin, Spermidine, Spermine, Diacetylspermine, Taurine, Tyramine |
| Amine Oxide | 1 | Trimethylamine N-oxide (TMAO) |
| Carboxylic Acid | 1 | Homocysteine |
| Monosaccharides | 1 | Glucose |
| Organic Acids | 17 | Lactic acid, Beta-hydroxybutyric acid, Alpha-ketoglutaric acid, Citric acid, Butyric acid, Propionic acid, HPHPA, Para-hydroxyhippuric acid, Succinic acid, Fumaric acid, Pyruvic acid, Isobutyric acid, Hippuric acid, Methylmalonic acid, Homovanillic acid, Indole acetic acid, Uric acid |
| Diacyl-phosphatidylcholines | 8 | PC aa C32:2/ C36:0/ C36:6/ C38:0/ C38:6/ C40:1/ C40:2/ C40:6 |
| Acyl-alkyl- phosphatidylcholines | 2 | PC ae C36:0/ C40:6 |
| Lyso-phosphatidylcholines | 14 | LysoPC a C14:0/ C16:0/ C16:1/ C17:0/ C18:0/ C18:1/ C18:2/ C20:3/ C20:4/ C24:0/ C26:0/ C26:1/ C28:0/ C28:1 |
| Sphingomyelines | 5 | SM C16:0, SM C16:1, SM C18:0, SM C18:1, SM C20:2 |
| Hydroxysphingomyelines | 5 | SM (OH) C14:1, SM (OH) C16:1, SM (OH) C22:1, SM (OH) C22:2, SM (OH) C24:1 |
| Vitamins & Cofactors | 1 | Choline |

C: carbon; DC: decarboxyl; M: methyl; OH: hydroxyl; TMAO: Trimethylamine N-oxide; HPHPA: 3-(3-hydroxyphenyl)-3-hydroxypropionic acid; PC: phophatidylcholine; aa: acyl-acyl; ae, acyl-alkyl; lysoPC: lysophosphatidylcholine; SM: sphingomyelin.
