# Supplementary material for: Metabolomic signatures for the longitudinal reduction of muscle strength over 10 years
Source: Skelet Muscle. 2022 Feb 7;12:4. doi: 10.1186/s13395-022-00286-9 (PMC8819943; doi:10.1186/s13395-022-00286-9)
Supplement: Supplementary file 2 — Additional file 2: Supplementary table 2: Summary statistics for the 129 metabolites that passed the quality checks and were included in the analysis. [file 13395_2022_286_MOESM2_ESM.docx]

**Supplementary table 2:** Summary statistics for the 129 metabolites that passed the quality checks and were included in the analysis.

| **#** | **Metabolites** | **Mean±SD** | **Min** | **Max** |
| --- | --- | --- | --- | --- |
| 1 | Creatinine | 95.25±30.52 | 27.7 | 212 |
| 2 | Glycine | 323.39±96.86 | 161 | 892 |
| 3 | Alanine | 496.25±113.63 | 268 | 970 |
| 4 | Serine | 136.28±25.42 | 68.5 | 229 |
| 5 | Proline | 222.82±65.68 | 85.4 | 516 |
| 6 | Valine | 267.14±52.76 | 139 | 465 |
| 7 | Threonine | 174.27±39.69 | 83.9 | 338 |
| 8 | Taurine | 113.89±35.71 | 46.4 | 237 |
| 9 | Putrescine | 0.26±0.13 | 0.0521 | 0.972 |
| 10 | trans-Hydroxyproline | 13.83±6.30 | 5.11 | 40.9 |
| 11 | Leucine | 173.90±40.50 | 92.7 | 317 |
| 12 | Isoleucine | 80.06±18.20 | 39.4 | 134 |
| 13 | Asparagine | 46.81±10.29 | 28.1 | 93.5 |
| 14 | Aspartic acid | 16.33±6.61 | 3.02 | 51.8 |
| 15 | Glutamine | 751.19±124.18 | 419 | 1230 |
| 16 | Glutamic acid | 58.72±25.80 | 12.8 | 164 |
| 17 | Methionine | 32.27±6.60 | 18.6 | 53.6 |
| 18 | Histidine | 109.68±29.18 | 62.6 | 511 |
| 19 | alpha-Aminoadipic acid | 0.71±0.36 | 0.0271 | 3.52 |
| 20 | Phenylalanine | 84.07±15.04 | 55.3 | 194 |
| 21 | Methionine-sulfoxide | 0.88±0.36 | 0.183 | 2.19 |
| 22 | Arginine | 156.79±33.97 | 88.2 | 283 |
| 23 | Acetyl-ornithine | 0.95±0.79 | 0.0669 | 10.5 |
| 24 | Citrulline | 44.24±12.34 | 9.75 | 141 |
| 25 | Asymmetric dimethylarginine | 0.72±0.76 | 0.331 | 14.3 |
| 26 | Serotonin | 1.27±0.73 | 0.0302 | 4.71 |
| 27 | Tyrosine | 90.09±22.16 | 45.1 | 170 |
| 28 | Kynurenine | 3.08±1.06 | 1.05 | 7.27 |
| 29 | Total dimethylarginine | 2.10±0.84 | 1.14 | 13.7 |
| 30 | Tryptophan | 75.71±15.32 | 29.1 | 131 |
| 31 | Ornithine | 112.97±26.41 | 52 | 217 |
| 32 | Lysine | 257.92±61.44 | 135 | 444 |
| 33 | Sarcosine | 1.78±0.84 | 0.203 | 7.21 |
| 34 | Spermidine | 0.18±0.07 | 0.0249 | 0.458 |

| **#** | **Metabolites** | **Mean±SD** | **Min** | **Max** |
| --- | --- | --- | --- | --- |
| 35 | Spermine | 0.13±0.04 | 0.0498 | 0.436 |
| 36 | Creatine | 42.33±22.19 | 8.79 | 131 |
| 37 | Betaine | 42.19±15.47 | 12.2 | 107 |
| 38 | Choline | 12.29±3.40 | 4.87 | 27 |
| 39 | Trimethylamine N-oxide | 8.86±9.57 | 0.432 | 93.6 |
| 40 | Methylhistidine | 15.92±10.69 | 3.72 | 70.8 |
| 41 | Lactic acid | 1778.59±585.23 | 516 | 4100 |
| 42 | beta-Hydroxybutyric acid | 89.18±98.63 | 13.2 | 736 |
| 43 | alpha-Ketoglutaric acid | 8.46±2.42 | 4.06 | 29.3 |
| 44 | Citric acid | 144.94±43.19 | 66.4 | 382 |
| 45 | Butyric acid | 0.72±0.32 | 0.161 | 2.48 |
| 46 | Propionic acid | 2.20±14.62 | 0.13 | 297 |
| 47 | HPHPA | 0.18±0.21 | 0.0101 | 1.44 |
| 48 | Succinic acid | 2.04±0.49 | 1.1 | 3.64 |
| 49 | Fumaric acid | 1.29±0.40 | 0.579 | 3.12 |
| 50 | Pyruvic acid | 58.44±20.35 | 13.7 | 149 |
| 51 | Isobutyric acid | 0.60±0.19 | 0.201 | 1.87 |
| 52 | Hippuric acid | 6.12±5.09 | 0.59 | 51.6 |
| 53 | Methylmalonic acid | 0.19±0.14 | 0.0247 | 1.73 |
| 54 | Indole acetic acid | 2.34±1.83 | 0.255 | 19.6 |
| 55 | Uric acid | 341.78±82.41 | 144 | 572 |
| 56 | Glucose | 5234.08±991.52 | 3571 | 13425 |
| 57 | LysoPC a C14:0 | 2.90±0.94 | 1.0659 | 6.9832 |
| 58 | LysoPC a C16:0 | 74.76±19.26 | 36.1693 | 164.2629 |
| 59 | LysoPC a C16:1 | 3.03±1.16 | 0.9811 | 12.9972 |
| 60 | LysoPC a C17:0 | 1.86±0.70 | 0.5198 | 5.8315 |
| 61 | LysoPC a C18:0 | 22.87±6.76 | 9.0653 | 50.5419 |
| 62 | LysoPC a C18:1 | 18.17±6.72 | 6.8234 | 79.2064 |
| 63 | LysoPC a C18:2 | 22.07±8.21 | 7.3367 | 62.6617 |
| 64 | LysoPC a C20:3 | 2.85±1.02 | 0.9418 | 10.4587 |
| 65 | LysoPC a C20:4 | 4.54±1.68 | 1.6613 | 14.6625 |
| 66 | LysoPC a C24:0 | 0.10±0.03 | 0.0314 | 0.2355 |
| 67 | LysoPC a C26:0 | 0.11±0.04 | 0.0328 | 0.2671 |
| 68 | LysoPC a C26:1 | 0.05±0.02 | 0.0097 | 0.1206 |
| 69 | LysoPC a C28:0 | 0.21±0.07 | 0.0674 | 0.588 |
| 70 | LysoPC a C28:1 | 0.27±0.10 | 0.0621 | 0.6177 |
| 71 | SM C16:0 | 130.43±30.02 | 60.8714 | 231.5374 |
| 72 | SM C16:1 | 19.36±4.82 | 8.3687 | 37.9455 |
| **#** | **Metabolites** | **Mean±SD** | **Min** | **Max** |
| 73 | SM C18:0 | 32.40±8.14 | 15.4762 | 59.3029 |
| 74 | SM C18:1 | 14.07±4.20 | 5.185 | 28.7409 |
| 75 | SM C20:2 | 1.67±0.52 | 0.6583 | 4.0601 |
| 76 | SM (OH) C14:1 | 9.43±2.81 | 3.2538 | 18.4442 |
| 77 | SM (OH) C16:1 | 5.62±1.60 | 1.8069 | 11.2314 |
| 78 | SM (OH) C22:1 | 18.99±5.17 | 6.663 | 41.0485 |
| 79 | SM (OH) C22:2 | 17.04±4.74 | 6.7205 | 35.1672 |
| 80 | SM (OH) C24:1 | 2.84±0.79 | 1.0599 | 6.3808 |
| 81 | PC aa C32:2 | 6.74±1.95 | 2.4347 | 14.5156 |
| 82 | PC aa C36:0 | 6.85±1.92 | 2.8197 | 14.6563 |
| 83 | PC aa C36:6 | 1.20±0.51 | 0.275 | 4.2165 |
| 84 | PC aa C38:0 | 2.96±1.17 | 0.9397 | 8.4584 |
| 85 | PC aa C38:6 | 73.95±27.16 | 22.1437 | 224.4749 |
| 86 | PC aa C40:1 | 0.25±0.07 | 0.1151 | 0.5082 |
| 87 | PC aa C40:2 | 0.34±0.08 | 0.1573 | 0.6621 |
| 88 | PC aa C40:6 | 22.01±8.43 | 5.7374 | 62.3499 |
| 89 | PC ae C36:0 | 1.70±0.47 | 0.5595 | 3.4495 |
| 90 | PC ae C40:6 | 4.37±1.43 | 1.8528 | 9.777 |
| 91 | C0 | 54.08±13.14 | 21.6175 | 98.7145 |
| 92 | C2 | 12.02±4.22 | 3.172 | 30.0657 |
| 93 | C3 | 0.37±0.12 | 0.1308 | 1.0289 |
| 94 | C3:1 | 0.01±0.00 | 0.0017 | 0.0292 |
| 95 | C4 | 0.29±0.13 | 0.0801 | 1.2048 |
| 96 | C4:1 | 0.02±0.01 | 0.0062 | 0.1398 |
| 97 | C5 | 0.15±0.06 | 0.0586 | 0.4868 |
| 98 | C5:1 | 0.02±0.01 | 0.0077 | 0.065 |
| 99 | C6(or C4:1-DC) | 0.08±0.04 | 0.0276 | 0.5666 |
| 100 | C6:1 | 0.02±0.01 | 0.0073 | 0.048 |
| 101 | C8 | 0.25±0.22 | 0.0519 | 2.9411 |
| 102 | C9 | 0.08±0.05 | 0.0183 | 0.4074 |
| 103 | C10 | 0.57±0.46 | 0.1078 | 6.0561 |
| 104 | C10:1 | 0.32±0.12 | 0.125 | 0.9399 |
| 105 | C10:2 | 0.06±0.02 | 0.0267 | 0.1799 |
| 106 | C12 | 0.14±0.07 | 0.0439 | 0.8476 |
| 107 | C12:1 | 0.17±0.06 | 0.0685 | 0.5337 |
| 108 | C14 | 0.06±0.02 | 0.019 | 0.2495 |
| 109 | C14:1 | 0.20±0.09 | 0.0686 | 0.9702 |
| 110 | C14:2 | 0.06±0.03 | 0.0194 | 0.2514 |
| **#** | **Metabolites** | **Mean±SD** | **Min** | **Max** |
| 111 | C16 | 0.15±0.04 | 0.0504 | 0.3142 |
| 112 | C16:1 | 0.06±0.02 | 0.0257 | 0.2616 |
| 113 | C16:2 | 0.02±0.01 | 0.0067 | 0.0674 |
| 114 | C18 | 0.06±0.02 | 0.0276 | 0.1372 |
| 115 | C18:1 | 0.19±0.06 | 0.0581 | 0.4725 |
| 116 | C18:2 | 0.06±0.02 | 0.0164 | 0.1283 |
| 117 | C3-OH | 0.03±0.01 | 0.0098 | 0.0647 |
| 118 | C4-OH(or C3-DC) | 0.04±0.02 | 0.0163 | 0.1363 |
| 119 | C5-OH(or C3-DC-M) | 0.03±0.01 | 0.0118 | 0.081 |
| 120 | C5-DC(or C6-OH) | 0.01±0.01 | 0.0034 | 0.0356 |
| 121 | C5:1-DC | 0.03±0.01 | 0.011 | 0.0789 |
| 122 | C5M-DC | 0.03±0.01 | 0.0109 | 0.1407 |
| 123 | C12-DC | 0.01±0.00 | 0.0041 | 0.016 |
| 124 | C14:1-OH | 0.03±0.01 | 0.0121 | 0.0731 |
| 125 | C14:2-OH | 0.02±0.00 | 0.0053 | 0.0383 |
| 126 | C16-OH | 0.01±0.00 | 0.0041 | 0.0247 |
| 127 | C16:1-OH | 0.02±0.01 | 0.0084 | 0.047 |
| 128 | C16:2-OH | 0.01±0.00 | 0.0055 | 0.0243 |
| 129 | C18:1-OH | 0.01±0.00 | 0.0048 | 0.0368 |

C: carbon; DC: decarboxyl; M: methyl; OH: hydroxyl; TMAO: Trimethylamine N-oxide; HPHPA: 3-(3-hydroxyphenyl)-3-hydroxypropionic acid; PC: phophatidylcholine; aa: acyl-acyl; ae, acyl-alkyl; lysoPC: lysophosphatidylcholine; SM: sphingomyelin.
