# Supplementary material for: Metabolomic signatures for the longitudinal reduction of muscle strength over 10 years
Source: Skelet Muscle. 2022 Feb 7;12:4. doi: 10.1186/s13395-022-00286-9 (PMC8819943; doi:10.1186/s13395-022-00286-9)
Supplement: Supplementary file 3 — Additional file 3: Supplementary table 3: Top metabolites associated with the muscle strength change measures in three different age groups over 10-years follow up time. [file 13395_2022_286_MOESM3_ESM.docx]

**Supplementary table 3:** Top metabolites associated with the muscle strength change measures in three different age groups over 10-years follow up time.

|  |  | (Metabolite*follow-up time)^‡^ | **Beta** | **SE** | **P-value** |
| --- | --- | --- | --- | --- | --- |
| **Hand Grip** | **Younger Age Group** | Asymmetric dimethylarginine | -0.049 | 0.13 | 0.69 |
|  |  | Total dimethylarginine | -0.01 | 0.05 | 0.79 |
|  | **Middle Age Group** | Asymmetric dimethylarginine | -0.056 | 0.01 | 0.0005 |
|  |  | Total dimethylarginine | -0.05 | 0.02 | 0.003 |
|  | **Older Age Group** | Asymmetric dimethylarginine | -0.13 | 0.12 | 0.29 |
|  |  | Total dimethylarginine | -0.03 | 0.04 | 0.56 |
|  |  |  |  |  |  |
| **Knee Extension** | **Younger Age Group** | Asymmetric dimethylarginine | -0.51 | 0.41 | 0.22 |
|  |  | Total dimethylarginine | -0.06 | 0.16 | 0.70 |
|  | **Middle Age Group** | Asymmetric dimethylarginine | -0.10 | 0.05 | 0.03 |
|  |  | Total dimethylarginine | -0.1 | 0.05 | 0.06 |
|  | **Older Age Group** | Asymmetric dimethylarginine | -0.60 | 0.34 | 0.075 |
|  |  | Total dimethylarginine | -0.21 | 0.12 | 0.083 |
|  |  |  |  |  |  |
| **Leg Strength** | **Younger Age Group** | Uric acid | -0.64 | 0.26 | 0.02 |
|  | **Middle Age Group** | Uric acid | -0.73 | 0.3 | 0.01 |
|  | **Older Age Group** | Uric acid | -0.52 | 0.26 | 0.05 |

^‡^An interaction term between metabolite values and follow-up time used as predictors for longitudinal changes in muscle strength.
